# Balloon Angioplasty for Recoarctation in Infants with Isolated Aortic Coarctation and Coarctation Associated with Other Congenital Heart Diseases

**DOI:** 10.3390/jcdd13090473

**Published:** 2026-09-20

**Authors:** Axel Rentzsch, Sophie Schubert, Jochen Pfeifer, Migdat Mustafi, Hashim Abdul-Khaliq

**Affiliations:** 1 Clinic for Pediatric Cardiology, Saarland University Hospital, 66421 Homburg, Germany; 2 Department of Pediatrics, Clinic Donaustadt, 1220 Vienna, Austria; 3 Department of Thoracic and Cardiovascular Surgery, Saarland University Hospital, 66421 Homburg, Germany

**Keywords:** early balloon angioplasty, recoarctation, aortic coarctation, congenital heart disease

## Abstract

Background: For infants who underwent surgical coarctation repair and required balloon angioplasty for recoarctation within the first year of life, data are limited—especially regarding safety, mid-term efficacy, and reintervention risk factors. Methods: This retrospective study included the data of 33 infants who underwent surgical correction of CoA within their first three months of life and whose first cardiac catheterization for recoarctation—defined by a systolic echocardiographic gradient of ≥20 mmHg—was performed within the first year of life. Results: The median age at repair was 21 days, and that at angioplasty was 77 days. The peak systolic gradient decreased from 40.0 to 21.0 mmHg (*p* < 0.001), and the mean gradient decreased from 13.9 to 6.7 mmHg (*p* = 0.02). The aortic stenotic diameter increased from 3.2 to 4.1 mm (*p* < 0.001), and the z score increased from −4.2 to −2.7 (*p* < 0.001). Right arm systolic blood pressure dropped from 111.8 to 88.2 mmHg (*p* < 0.001); 7% of cases remained hypertensive. Complications occurred in 12% of patients, and reintervention was needed in 39.4%. Association with other congenital heart disease increased reintervention risk (OR 4.51). Follow-up gradients were as follows: 27.4 ± 10.2 mmHg (1 year), 24.6 ± 8.3 mmHg (2 years), 32.8 ± 18.8 mmHg (5 years), and 35.4 ± 23.2 mmHg (7 years). Conclusions: Early balloon angioplasty for postoperative recoarctation is safe, effective, and repeatable, with a high procedural success rate and sustained hemodynamic improvements. Reinterventions were more common in patients with additional CHD, though outcomes remained favorable.

## 1. Introduction

Aortic coarctation (CoA) is a congenital heart defect characterized by narrowing of the aorta between the distal aortic arch and the descending aorta. The severity of the stenosis determines the clinical presentation of the disease, which can range from asymptomatic cases to acute cardiac decompensation in severely affected infants [1]. Hemodynamically significant stenoses in neonates and infants are typically corrected surgically due to the high rate of restenosis associated with primary catheter-based interventions in this age group. Surgical correction has significantly improved overall survival for these patients; however, restenosis remains a common complication, with reported rates ranging from 6% to 42% in infants [2,3,4]. Balloon angioplasty is a safe and effective treatment option for postoperative restenosis in children and adolescents [5]. Stent implantation has also yielded favorable results for both native and recurrent coarctation [6,7,8]. However, its use in infants poses several challenges, including limited growth adaptability, in-stent restenosis, and the need for subsequent dilatation or additional surgical interventions. In early restenosis after operation, however, the utility of stent implantation remains less well defined, as the elasticity of the vessel wall and the potential for vascular injury have raised concerns about long-term efficacy and complications. While previous studies have demonstrated the feasibility of balloon angioplasty in this setting, data on its effectiveness, safety, mid-term efficacy and risk factors in young infants remain limited [3,9,10,11].

The present study specifically addresses the limited evidence regarding balloon angioplasty as the sole initial intervention for early postoperative recoarctation in infants under one year of age. The analysis focuses on procedural success, complication rates, restenosis risk, and medium-term outcomes in this vulnerable population. These findings may help to better define the role of early balloon angioplasty in current treatment strategies and to identify predictive factors for reintervention.

## 2. Materials and Methods

### 2.1. Study Design

This retrospective, single-center study was conducted at Saarland University Medical Center, Homburg, Germany, between November 1994 and August 2022. Patient data were retrieved from the institutional database and the patients’ electronic medical records. The study was approved by the local ethics committee (approval ID: 55/25).

### 2.2. Patient Population

During the study period, 146 patients underwent primary surgical correction of CoA (mean age 0.98 years, SD 2.63 years). Of these, 103 did not develop recoarctation, while 43 did (29.4%). The present study included 33 infants from this group who underwent surgical correction within their first three months of life and required their first cardiac catheterization for recoarctation—defined by a systolic echocardiographic gradient of at least 20 mmHg—within the first year of life. This echocardiographic threshold served as a screening criterion to identify candidates for catheterization; the diagnosis of recoarctation was confirmed by the invasively measured gradient at the time of the procedure. The remaining 10 patients with recoarctation were treated beyond the first year of life (mean age 8.9 years). Fifteen patients of the study group had isolated CoA, while 18 presented with CoA and additional congenital heart disease (CHD) lesions, defined as CoA in combination with at least one additional hemodynamically relevant congenital heart defect (e.g., ventricular septal defect or hypoplastic aortic arch). Patients with isolated CoA had only minor additional findings, such as bicuspid aortic valve or hemodynamically irrelevant septal defects. All data analyzed in this study were retrospectively obtained from patients’ medical records.

### 2.3. Non-Invasive Cardiovascular Assessment

The echocardiographic assessment focused on the maximal systolic pressure gradient across the aortic arch and was performed using Doppler echocardiography within two weeks before and after balloon angioplasty. At the same time, systemic blood pressure was measured in a calm, resting state. During medium-term follow-up, echocardiographic examinations were conducted at 1, 2, 5, and 7 years.

### 2.4. Intervention: Balloon Angioplasty Procedure

Balloon angioplasty was carried out under general anesthesia or deep sedation by a single experienced interventional cardiologist. To minimize thromboembolic complications, patients received an intravenous, weight-adjusted heparin dose of 100 IU/kg of body weight. Peri-interventional antibiotic prophylaxis with a cephalosporin was administered. Vascular access was obtained via the femoral artery, and a 4F or 5F arterial sheath was inserted under sterile conditions. Baseline hemodynamic measurements, including pressure values proximal and distal to the site of stenosis and the peak systolic pressure gradient, were recorded. Angiography was performed to assess the anatomical characteristics of the aortic arch and descending aorta, with measurements taken at the transverse aortic arch and at the site of the stenosis before and after dilation.

**Figure 1:**
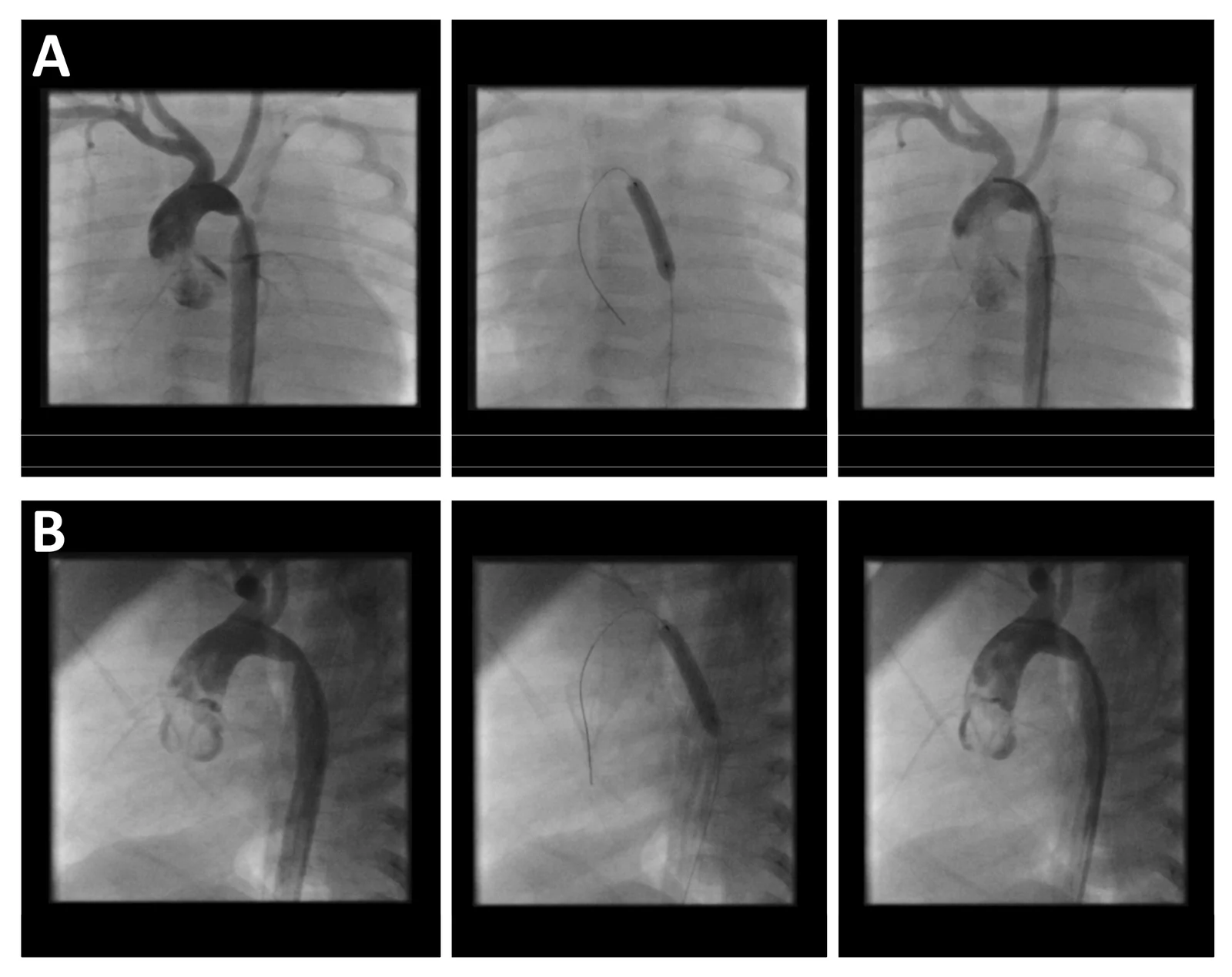
Representative angiographic images of a 2-month-old patient with recurrent coarctation of the aorta, obtained one month after surgical repair. Images were acquired before (**left**), during (**middle**), and after (**right**) balloon angioplasty in posteroanterior (**A**) and lateral (**B**) projections, demonstrating an increase in vessel diameter at the site of restenosis.

Balloon selection—including type, length, and diameter—was based on the measured vessel diameter, the severity of the stenosis, and the operator’s clinical judgment. Low- and high-pressure balloons, such as the Maverick Monorail Coronary Balloon (Boston Scientific, Marlborough, MA, USA), Tyshak mini^®^-Valvuloplasty Balloon (Heart Medical, Best, The Netherlands), or VACS^®^ Dilatation Catheter (OSYPKA, Rheinfelden, Germany), were used and gradually inflated using a controlled stepwise approach. Typically, two to three inflations were performed per procedure using progressively larger balloons until significant stenosis reduction was achieved. Angiographic images of the aorta and hemodynamic parameters were obtained before and after balloon angioplasty (Figure 1).

Repeat angiographic imaging was performed to assess aortic wall integrity and exclude the development of complications such as dissection or rupture. After sheath removal and achievement of hemostasis, patients were monitored in the intensive care unit for at least 24 h with continuous hemodynamic monitoring, and intravenous heparin was routinely administered. All patients received low-dose aspirin (3–5 mg/kg/day) for at least three months.

### 2.5. Outcome Measures

All aortograms, cardiac catheterization reports, and echocardiographic examinations were retrospectively reviewed by an independent examiner, a pediatric interventional cardiologist with extensive experience in angiographic interpretation. The stenosis diameter was carefully measured digitally from the stored angiographic studies in both the posteroanterior (p.a.) and lateral (lat.) projections. The corresponding z score of the p.a. diameter was calculated according to Pettersen et al. [12], the balloon-to-stenosis ratio was determined, and the diameter of the transverse aortic arch was assessed.

Primary endpoints included the change in the peak systolic pressure gradient across the aortic isthmus, the stenotic segment diameter and its corresponding z score, and echocardiographic parameters assessed in the short and medium term.

Secondary endpoints comprised the need for additional balloon angioplasty or surgical revision and post-procedural hypertension. Additionally, the impact of an additional congenital heart defect on post-interventional outcomes was evaluated, focusing on its influence on restenosis risk and the need for further interventions.

### 2.6. Complications

Complications were defined as any adverse event occurring during or within 24 h after the balloon angioplasty procedure. Severe complications were classified as events resulting in permanent harm or requiring surgical intervention.

### 2.7. Statistical Analysis

Data analysis was performed using SPSS Version 29.0.2.0 (IBM Corp., Armonk, NY, USA). Descriptive statistics included the means, standard deviations, and frequency distributions. Pre- and post-interventional continuous variables were compared with the Wilcoxon signed-rank test because of their nonparametric nature. Categorical variables were analyzed using the chi-square test. The McNemar test was applied to assess changes in the proportions of patients with systolic blood pressure above the 95th percentile (age- and height-adjusted). The Friedman test was used to evaluate changes in the echocardiographic peak systolic gradients across multiple follow-up points. Associations between potential predictive factors and the need for reintervention were assessed using logistic regression analysis for continuous variables and the chi-square test for categorical variables. Odds ratios (ORs) were calculated using logistic regression. A *p* value < 0.05 indicated statistical significance.

## 3. Results

We identified a total of 33 patients (17 females, 51.5%; 16 males, 48.5%) who underwent primary surgical repair of CoA within the first 3 months of life and required balloon angioplasty for recoarctation within the first year of life. The median age at the time of primary surgical repair was 21 days (range: 3–74 days). Surgical correction was performed via resection of the aortic isthmus stenosis with end-to-end anastomosis in 30 patients (90.9%), one patient (3.0%) underwent patch aortoplasty due to severe hypoplasia of the aortic arch, and two patients (6.1%) underwent a Damus–Kaye–Stansel procedure with reconstruction of the aortic arch, including the isthmus, due to complex univentricular heart disease (double-inlet left ventricle, hypoplastic right ventricle, and transposition of the great arteries).

The first balloon angioplasty was performed at a median age of 77 days (range: 16–352 days), with patients weighing a mean of 4.9 kg (range: 2.1–9.1 kg). The median interval between initial surgical repair and subsequent balloon angioplasty was 60 days (range: 5–339 days). No statistically significant differences were found regarding sex distribution at the time of surgery or first balloon angioplasty. Among the 33 patients, 18 (54.5%) had CoA with additional CHD lesions (Table 1).

### 3.1. Procedural Results

**Figure 2:**
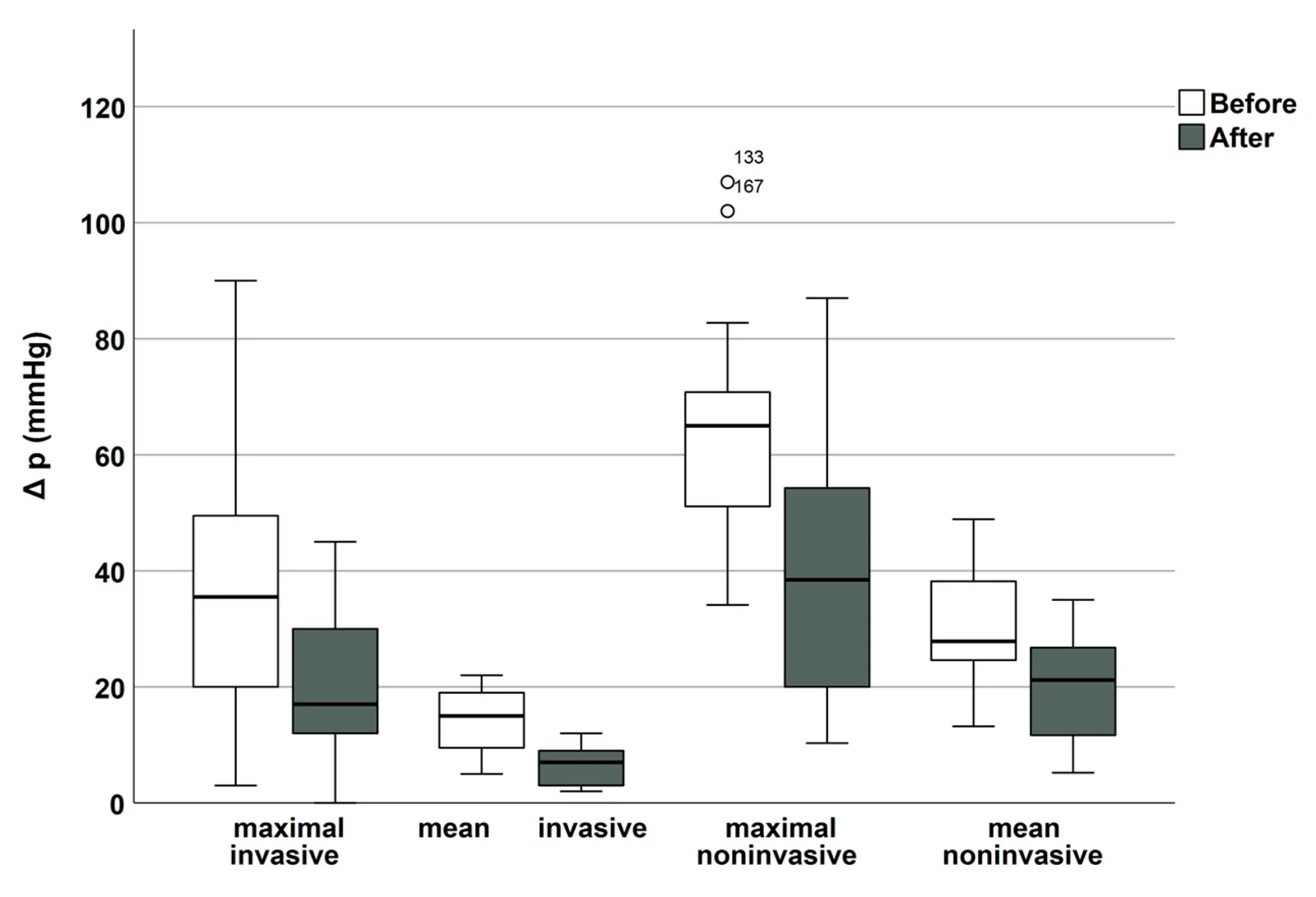
Boxplots of systolic pressure gradients before and after balloon angioplasty, measured invasively and non-invasively.

**Figure 3:**
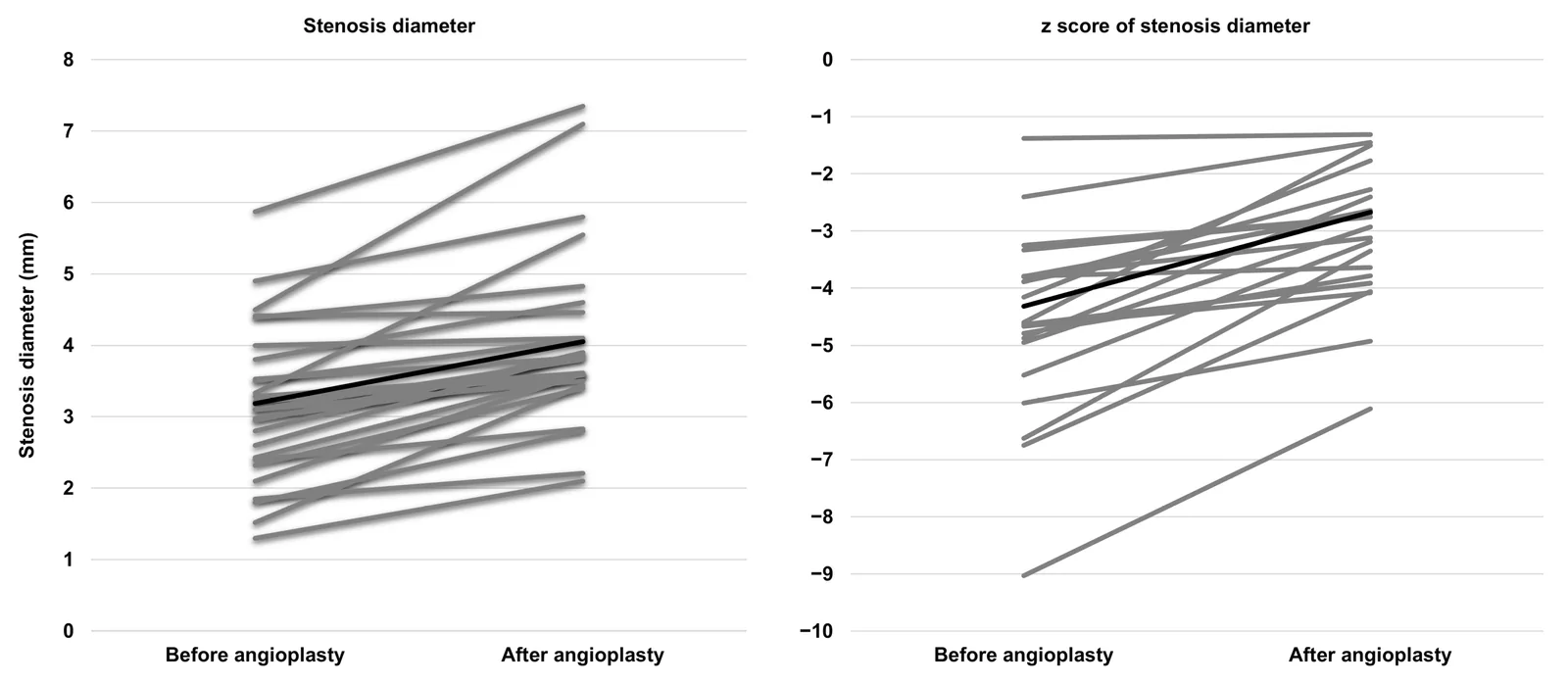
Individual changes in stenosis diameter (**left**) and the corresponding z score (**right**) before and after balloon angioplasty. Each line represents the data of one patient. Black lines indicate the mean values.

The echocardiographic, hemodynamic, and anatomical parameters of the patients before and after balloon angioplasty are summarized in Table 2. Significant improvements were observed in all key measurements, including echocardiographic and invasive pressure gradients, stenosis diameter and systolic blood pressure, and the arm–leg pressure gradient (Figure 2 and Figure 3). Notably, echocardiographic gradients were consistently higher than invasively measured values.

### 3.2. Reinterventions, Predictive Factors, and Complications

A total of 39.4% of patients (13 out of 33) required at least one additional balloon angioplasty due to recurrent restenosis at a mean age of 3.0 ± 3.9 years (range 0.2–12.0 years). Among them, eight patients (24.2%) further required a third catheterization, whereas four patients (12.1%) required a fourth intervention. Three patients (9%) underwent stent implantation in the isthmic region during follow-up. Three of 33 patients (9%) required reoperation involving the aortic arch after one redilatation: one with isolated CoA, one with tricuspid atresia who underwent aortic arch augmentation during partial cavopulmonary connection placement, and one with double-outlet right ventricle and Taussig–Bing anomaly during corrective surgery.

Analysis of the potential predictive factors for reinterventions revealed that a larger z score of the stenosis diameter after the initial balloon dilation was associated with a lower likelihood of reintervention (odds ratio (OR) 0.35, *p* = 0.003). Similarly, patients with a larger stenotic diameter z score before the initial balloon dilation were also less likely to need additional procedures (OR 0.55, *p* = 0.012). The presence of associated CHD lesions increased the risk of reintervention (OR 4.51, *p* = 0.13), with 50.0% of patients harboring such defects requiring a second dilation compared with 26.7% of those with isolated CoA; however, this association did not reach statistical significance. Patient age, body weight, balloon-to-stenosis ratio, initial systolic pressure gradient, and diameter of the transverse aortic arch were not significantly associated with the likelihood of reintervention.

Regarding complications of the initial intervention, 4 out of 33 patients (12.1%) experienced a catheter-associated event. One patient experienced post-procedural bleeding at the puncture site, which was controlled with protamine and erythrocyte concentrate due to a drop in hemoglobin. Another developed femoral artery stenosis, resulting in transient circulatory disturbances in the punctured leg; these resolved with intravenous heparin and iloprost therapy. A third patient was diagnosed with necrotizing enterocolitis immediately after the intervention, with symptoms improving within a few days under conservative treatment. Finally, one patient experienced a second-degree AV block, which resolved within an hour following orciprenaline administration. No severe complications like aortic dissection, aneurysm formation, or death were observed during follow-up.

### 3.3. Follow-Up

As a measure of long-term treatment efficacy, echocardiographic peak systolic pressure gradients were assessed at multiple follow-up time points. Follow-up data were available for 17/33 (1 year), 14/33 (2 years), 14/33 (5 years), and 13/33 (7 years) patients. After one year, the mean peak systolic gradient was 27.4 ± 10.2 mmHg; this gradient slightly decreased to 24.6 ± 8.3 mmHg at two years but increased to 32.8 ± 18.8 mmHg at five years, and a further increase to 35.4 ± 23.2 mmHg was observed at seven years. Although no statistically significant differences were observed, mean peak systolic gradients showed a gradual increase from the two-year to the seven-year follow-up.

## 4. Discussion

This study aimed to evaluate the safety and efficacy of balloon angioplasty for residual CoA in infants. A substantial proportion of infants develop early restenosis after surgical correction of CoA—29% of those who underwent repair within the first three months of life in our cohort—thus requiring an intervention to reduce left ventricular afterload. Our data suggest that balloon angioplasty may be considered a safe and effective treatment option for restenosis following primary surgical repair of CoA in infancy. The significant reduction in systolic pressure gradients—measured both invasively and by echocardiography—and the increase in stenotic diameter indicates a substantial procedural success rate. It should be noted that echocardiographic pressure gradients are consistently higher than invasively measured values, as they reflect the maximum instantaneous gradient obtained in the awake child, whereas invasive measurements represent the peak-to-peak gradient under sedation. Although the post-interventional echocardiographic gradient remained above the threshold used to define recoarctation, this reflects a conservative dilation approach in infants to minimize the risk of aortic or arterial access injury. Whether a more aggressive dilation approach would have further reduced residual gradients without increasing complications remains an open question that warrants further investigation. While these findings were observed in a particularly young cohort, as all included patients were under one year of age, they are consistent with previous studies reporting the efficacy of balloon dilation in postoperative recoarctation [11,14,15]. Thus, this study provides targeted insights into an age group that has been only marginally represented in the literature.

### 4.1. Reintervention Rates

This study’s reintervention rate of 39.4% is comparable to rates reported in the literature, such as 28% by Reich et al. [15] and 37% by Siblini et al. [16]. This relatively high rate reflects our study design: we included only infants who underwent CoA repair within the first three months of life and required balloon angioplasty for recoarctation within the first year. In contrast, many comparable studies included older patients and longer follow-up periods, thereby increasing the overall cohort size, which could have reduced the proportion of patients requiring reintervention. In this study, the rate of reintervention was higher among patients with CoA associated with other CHD than among those with isolated CoA (55.6% vs. 26.7%), consistent with findings reported elsewhere [17,18]. Although this difference did not reach statistical significance, it may suggest a clinically relevant association between anatomical complexity and the likelihood of repeated intervention. Agnoletti et al. [17] demonstrated that recoarctation occurs more frequently and earlier in CoA patients with associated cardiac anomalies, possibly due to hemodynamic conditions such as increased pulmonary blood flow (e.g., in VSD) or more extensive ductal tissue in complex anatomies. Notably, both pre- and post-interventional stenosis diameters (z scores) were significantly associated with reintervention risk, contrasting with previous studies that identified pressure gradients as the primary predictor [14,15,19]. This discrepancy may relate to the presence of ductal tissue in the coarctation shelf, which lacks stabilizing elastic structures and is prone to recoil [20,21,22].

### 4.2. Therapeutic Options for Re-CoA

Surgical correction remains the standard approach in neonates and infants. Despite promising approaches using stent implantation for primary CoA correction [23,24], registry data indicate that the need for redo procedures after primary interventional treatment ranges from 36% to 44% across all age groups, compared to 21% to 24% after initial surgical repair. Notably, the highest rates of redo procedures occur within the first year following interventional correction [25].

Regarding re-coarctation therapy, there is still no direct comparative evidence to guide the choice between balloon dilatation and stent implantation, although stent implantation in younger children has shown promising short-term outcomes [6,26]. Boe et al. reported reintervention rates after stent implantation as high as 72%, with a smaller sample size being associated with earlier reintervention [6]. Given the lack of growth-adapted stents and inconsistent evidence for their use in low-weight infants, balloon angioplasty—even if repeated—remains a safe and effective option for recoarctation in this group. Additionally, intimal proliferation may further limit the long-term efficacy of stent implantation in infants.

Reoperation may be required in selected cases, particularly when catheter-based approaches are not feasible due to complex anatomy (e.g., Shone’s syndrome, long-segment hypoplasia) or when concurrent surgical correction of associated lesions is necessary. For postsurgical recoarctation, catheter-based reintervention is the intervention of choice in older children, it is less invasive and is associated with shorter hospital stays [27]. Gendera et al. reported in a cohort of 401 patients that catheter-based reintervention was performed nearly twice as often as reoperation (9.7% vs. 4.5%) [28]. Notably, they identified operation in the neonatal period (<28 days) as an independent risk factor for catheter-based reintervention, which aligns with our cohort characteristics (median age at repair 21 days). In our series, 9% of patients required reoperation.

### 4.3. Complications

The complication rate of 12.1% is within the range reported by comparable studies, and all identified complications were transient, causing no permanent harm [11,29]. The absence of severe complications, such as aortic dissection or aneurysmal changes, underscores the safety of the procedure. Numerous studies have demonstrated that balloon angioplasty can be performed safely in children of different ages [5,30,31]. The single case of necrotizing enterocolitis seen in our study is most likely attributable to significant hypoperfusion of the lower body, including the intestinal organs, as described in patients with congenital heart defects with systemic outflow obstruction [32,33]. Given the low rate of transient, minor complications, this approach may be justified in light of the potential to prevent stent implantation or even reoperation.

### 4.4. Post-Procedural Hypertension

Balloon dilatation led to a significant reduction in systolic blood pressure and the arm–leg gradient. However, 7% of patients remained hypertensive post-intervention, consistent with the approximately 20% prevalence of residual hypertension reported in CoA patients even without restenosis [34,35,36,37]. This persistence may reflect impaired elastic properties of the aorta, as demonstrated in previous studies [38,39].

### 4.5. Limitations

This study has several limitations. The small sample size (n = 33) restricts the statistical power, particularly for subgroup analyses, and limits the detection of subtle differences or risk factors. The retrospective, single-center design introduces potential biases, as treatment decisions and data collection were based on clinical practice rather than a standardized protocol. The long observation period, during which procedural techniques and equipment evolved, potentially introduced temporal confounding. Additionally, follow-up data were incomplete, which limits the assessment of long-term outcomes.

## 5. Conclusions

Our findings demonstrate that early balloon angioplasty for recoarctation after surgical repair of aortic coarctation in infants under one year of age achieves high procedural success and marked hemodynamic improvement with a low complication rate. These data suggest that serial balloon dilatation may be considered a safe and effective treatment option for recoarctation in this vulnerable age group, potentially avoiding the need for stent implantation in infancy. Future research should focus on prospective studies to refine patient selection, optimize timing of intervention, and assess whether stent-based strategies might offer advantages. In summary, repeated balloon angioplasty may be considered a low-risk strategy for early recoarctation, as it avoids stent implantation or reoperation while accepting infrequent, minor complications.

## Figures and Tables

**Table 1:** Associated cardiac malformations in the 18 patients with associated CHD.

Associated Malformation	*n*	Proportion (%)
Ventricular septal defect	13	72
Transposition of the great arteries	6	33
Aortic stenosis	4	22
Double-inlet left ventricle	3	17
Taussig–Bing anomaly	2	11
Shone’s complex	2	11
Hypoplastic left ventricle	2	11
Hypoplastic right ventricle	2	11
Double aortic arch	1	6
Tricuspid atresia	1	6
Double-outlet right ventricle	1	6

**Table 2:** Hemodynamic and anatomical parameters before and after balloon angioplasty. Values are presented as the means ± standard deviations or percentages and fractions where indicated. The minimal stenosis diameter was measured absolutely (in mm) and as a z score, both in the posteroanterior (p.a.) and lateral (lat.) projections. The >95th percentile for systolic blood pressure is defined according to reference values adjusted for patient age and height [13].

Parameter	Before Intervention	After Intervention	*p* Value
Echocardiographic maximum pressure gradient (mmHg)	62.2 ± 18.7	40.5 ± 20.1	<0.001
Echocardiographic mean pressure gradient (mmHg)	30.4 ± 9.2	19.8 ± 8.9	<0.001
Invasive maximum systolic pressure gradient (mmHg)	40.0 ± 20.0	21.0 ± 12.5	<0.001
Invasive mean systolic pressure gradient (mmHg)	14.0 ± 5.8	6.9 ± 3.4	0.02
Stenosis diameter (mm), p.a.	3.2 ± 1.1	4.1 ± 1.3	<0.001
Stenosis diameter z score, p.a.	−4.3 ± 1.9	−2.7 ± 1.5	<0.001
Stenosis diameter (mm), lat.	3.4 ± 1.9	4.3 ± 1.9	<0.001
Systolic blood pressure right arm (mmHg)	111.8 ± 16.2	88.2 ± 16.4	<0.001
Arm–leg systolic blood pressure gradient	30.8 ± 19.2	14.7 ± 10.2	<0.001
Systolic blood pressure > 95th percentile (%, n/total)	80% (24/30)	7% (2/28)	<0.001

## Data Availability

The data underlying the present study are available upon request from the corresponding author.

## Abbreviations

The following abbreviations are used in this manuscript:

CHD	congenital heart disease
CoA	aortic coarctation
F	French
IU	international unit
lat.	lateral
OR	odds ratio
p.a.	posteroanterior
